# *Plasmodium falciparum* Parasite Lines Expressing DC8 and Group A PfEMP1 Bind to Brain, Intestinal, and Kidney Endothelial Cells

**DOI:** 10.3389/fcimb.2022.813011

**Published:** 2022-01-28

**Authors:** Luana S. Ortolan, Marion Avril, Jun Xue, Karl B. Seydel, Ying Zheng, Joseph D. Smith

**Affiliations:** ^1^ Center for Global Infectious Disease Research, Seattle Children’s Research Institute, Seattle, WA, United States; ^2^ Department of Bioengineering, University of Washington, Seattle, WA, United States; ^3^ Department of Osteopathic Medical Specialties, College of Osteopathic Medicine, Michigan State University, East Lansing, MI, United States; ^4^ Blantyre Malaria Project, Kamuzu University of Health Sciences, Blantyre, Malawi; ^5^ Department of Pediatrics, University of Washington, Seattle, WA, United States

**Keywords:** *Plasmodium falciparum*, malaria, cytoadhesion, endothelial protein C receptor, kidney endothelial cell, intestinal endothelial cell

## Abstract

Cytoadhesion of *Plasmodium falciparum*-infected red blood cells is a virulence determinant associated with microvascular obstruction and organ complications. The gastrointestinal tract is a major site of sequestration in fatal cerebral malaria cases and kidney complications are common in severe malaria, but parasite interactions with these microvascular sites are poorly characterized. To study parasite tropism for different microvascular sites, we investigated binding of parasite lines to primary human microvascular endothelial cells from intestine (HIMEC) and peritubular kidney (HKMEC) sites. Of the three major host receptors for *P. falciparum*, CD36 had low or negligible expression; endothelial protein C receptor (EPCR) had the broadest constitutive expression; and intercellular adhesion molecule 1 (ICAM-1) was weakly expressed on resting cells and was strongly upregulated by TNF-α on primary endothelial cells from the brain, intestine, and peritubular kidney sites. By studying parasite lines expressing *var* genes linked to severe malaria, we provide evidence that both the DC8 and Group A EPCR-binding subsets of the *P. falciparum* erythrocyte membrane protein 1 (PfEMP1) family encodes binding affinity for brain, intestinal, and peritubular kidney endothelial cells, and that DC8 parasite adhesion was partially dependent on EPCR. Collectively, these findings raise the possibility of a brain-gut-kidney binding axis contributing to multi-organ complications in severe malaria.

## Introduction

The distinctive virulence of *Plasmodium falciparum* is caused in part through the cytoadhesion of *P. falciparum*-infected erythrocytes (IEs) in the microcirculation of different organs. Extensive sequestration of *P. falciparum*-IEs obstructs blood flow and can promote localized inflammation and organ dysfunction (Miller et al., 2002). The best studied examples are cerebral malaria, associated with high sequestered parasite burdens in the brain microcirculation (Marchiafava and Bignami, 1892; MacPherson et al., 1985; Taylor et al., 2004), and placental malaria, associated with high parasite burdens in the placental intervillous blood circulation (Brabin et al., 2004). However, mature forms of *P. falciparum*-IEs have broad sequestration in diverse microvascular beds, including the brain, gastrointestinal tract, subcutaneous adipose tissue of the skin, heart, lung, spleen and to a lesser extent, the kidney (Spitz, 1946; MacPherson et al., 1985; Milner et al., 2014; Milner et al., 2015), but parasite tropism for most organ sites remains poorly characterized.

Whereas the kidney is not a major site of parasite sequestration (Spitz, 1946; Milner et al., 2014), kidney injury is common in severe malaria. For instance, recent evidence indicates that acute kidney injury (AKI) is a common complication in African children with severe malaria and is associated with increased morbidity and mortality (Conroy et al., 2016; Sypniewska et al., 2017; Conroy et al., 2019; Batte et al., 2021). Moreover, renal impairment occurs in up to 37% of adult severe malaria cases and increased the risk of death by 4-fold (Dondorp et al., 2008b). In a large autopsy series, renal failure was characterized by acute tubular necrosis with accumulation of host monocytes and *P. falciparum*-IEs in the kidneys, especially in the peritubular capillary microcirculation (Nguansangiam et al., 2007). The gastrointestinal tract has been considered a relatively non-pathogenic sequestration site. However, it is a major site of parasite sequestration and there is increasing evidence that gastrointestinal barrier function may be compromised at higher parasite burdens (Wilairatana et al., 1997). For instance, the gastrointestinal tract was the most intense site of parasite sequestration in fatal pediatric CM cases (Seydel et al., 2012; Milner et al., 2014) and blocked capillaries in the rectal mucosa correlate to disease severity and metabolic acidosis in adult SM cases (Dondorp et al., 2008a). Moreover, acidic microbial products contribute to metabolic acidosis in adult severe malaria (Leopold et al., 2019) and children with severe malaria are at risk for invasive bacterial disease (Scott et al., 2011), suggesting compromise of gut integrity. Thus, while kidney and gut pathogenesis are not well understood, autopsy studies have led to the hypothesis that organ injury may result from common pathological processes, precipitated by cytoadherence of *P. falciparum*-IEs.

Cytoadhesion of *P. falciparum*-IEs is mediated by the *P. falciparum* erythrocyte membrane protein-1 (PfEMP1) family, encoded by a repertoire of about 60 *var* genes per parasite genotype (Baruch et al., 1995; Smith et al., 1995; Su et al., 1995). PfEMP1 proteins encode multiple adhesion domains, called Duffy binding-like (DBL) and cysteine-rich interdomain region (CIDR), which confer different binding properties (Smith et al., 2013). For instance, different subsets of PfEMP1 proteins encode binding activity for CD36 (Robinson et al., 2003; Hsieh et al., 2016), endothelial protein C receptor (EPCR) (Turner et al., 2013; Lau et al., 2015), and intercellular adhesion molecule 1 (ICAM-1) (Smith et al., 2000; Lennartz et al., 2019). Whereas the EPCR binders comprise only a small minority of the *var* gene repertoire (~10% of genes) (Rask et al., 2010), this subset is transcriptionally elevated in severe malaria infections and both the DC8 and Group A PfEMP1 variants are linked to severe malaria complications (Lavstsen et al., 2012; Turner et al., 2013; Bernabeu et al., 2016; Kessler et al., 2017; Lennartz et al., 2017; Mkumbaye et al., 2017; Sahu et al., 2021; Wichers et al., 2021). From *in vitro* studies, EPCR-binding variants adhere to human brain endothelial cells (Turner et al., 2013; Avril et al., 2016; Lennartz et al., 2017; Bernabeu et al., 2019; Storm et al., 2019) and to primary human heart and lung microvascular endothelial cells (Avril et al., 2013; Gillrie et al., 2015), suggesting they may have broad affinity for diverse microvascular beds.

Given that kidney injury and gastrointestinal sequestration are common in severe and fatal malaria cases, we investigated if similar parasite binding variants may have affinity for brain, intestinal, and kidney microvascular sites. Our analysis demonstrates that parasites expressing DC8 or Group A PfEMP1 encode broad binding affinity for primary human brain, intestinal, and kidney endothelial cells and are partially dependent on EPCR for adhesion.

## Materials and Methods

### Human Brain, Intestinal, and Peritubular Kidney Microvascular Endothelial Cell Cultures

Primary human brain microvascular endothelial cells (HBMEC) (Cell System, ACBRI 376) were cultured with endothelial cell growth basal medium-2 (EBM-2, Lonza, CC-3156) in accordance with manufacturer specifications with 5% fetal bovine serum (FBS) and supplements provided (Lonza, CC-4147) in tissue culture flasks treated with rat collagen I (Corning, 354236). Primary human intestinal microvascular endothelial cells (HIMEC) (Cell Systems, ACBRI 666) were cultured in complete classic medium with 10% FBS serum and culture boost (Cell Systems 4Z0-500) supplemented with Bac-off (Cell Systems) on an extracellular matrix-coated surface (attachment factor, Cell Systems). HBMEC and HIMEC were certified positive by the manufacturer for expression of Von Willebrand factor, acetylated low-density lipoprotein uptake, and CD31. HBMEC and HIMEC were used in experiments at passages 5 to 10. Primary human kidney peritubular microvascular endothelial cells (HKMEC) were isolated and purified from fetal kidneys as previously reported (Ligresti et al., 2016) and used in experiments at passages 3 to 5. Fetal human kidneys were obtained after informed consent from patients at the University of Washington Medical Center in compliance with Institutional Review Board protocol (IRB 447773EA). Cells were cultured and expanded on matrix-coated surface with 0.2% gelatin (Sigma, G1890) with EBM2 medium (Lonza, CC-3156) with 10% FBS and supplemented with 5 mg/ml EGCS (Cell Biologics, 1166), 1% antibiotic-antimycotic (Gibco, 15240062), 50 mg/ml of heparin (Sigma, H3149) and 20 ng/ml of vascular endothelial growth factor (VEGF) (R&D Systems, 293-VE-10).

### Characterization of Surface Markers on Endothelial Cells by Flow Cytometry

HBMEC, HIMEC and HKMEC monolayers were cultured on matrix-coated tissue culture flasks until confluence. Cells were washed with 1X PBS, lifted with 8 mM EDTA in 1X PBS and then resuspended in 1X PBS (supplemented with 2% FBS). Cell suspensions were stained with monoclonal antibodies (mAb) for CD31-PE (Biolegend, clone WM59), CD36-FITC (Biolegend, clone 5-271), EPCR-APC (Biolegend, clone RCR-401) and CD54/ICAM-1-PE-Cy-7 (Biolegend, clone HA58) at 1:100 dilution and with Live/Dead-V450 (Tonbo Biosciences) for 30 min on ice. For assays with proinflammatory pre-stimulation, confluent cell monolayers were stimulated with 10 ng/ml TNF-α (Sigma, T0157) for 20-24 hours at 37°C. Cells were analyzed in a LSRII (Becton & Dickson) with 100,000 events/sample. Gates were set based on fluorescence minus one (FMO) and IgG isotype controls (Biolegend). Results were expressed relative to IgG isotype controls. Data was analyzed using FlowJo v10 software (TreeStar Inc.).

### Immunofluorescence

HBMEC, HIMEC and HKMEC endothelial cells, were cultured in an 8 well pretreated chamber slide until confluence. For assays with proinflammatory pre-stimulation, confluent cell monolayers were stimulated with 10 ng/ml TNF-α (Sigma, T0157) for 24 hours at 5% CO_2_ and 37°C. Cells were fixed with 3.7% paraformaldehyde for 30 min. For antibody labeling, cells were pretreated with background blocking agent (Background Buster, Innovex Biosciences, NB306) for 30 min. Cells were then washed with 1x PBS and incubated with primary mouse-anti-human-ICAM-1 (1:200 dilution) in PBS (supplemented with 2% BSA) for 1 hour (Biosource International, ThermoFisher Scientific, clone C14), followed by the secondary antibody goat anti-mouse Alexa Fluor 488 (1:200 dilution) (Invitrogen) for 1 hour. Images taken under 400x magnification (Keyence BZ-X series microscope).

### *P. falciparum* Culture

The *P. falciparum* parasites lines IT4var19 (Avril et al., 2012), HB3var03 (Claessens et al., 2012; Avril et al., 2016), and 3173-S (Bernabeu et al., 2019) were cultured in human red blood cells (O+) and 10% pooled human A^+^ serum-rich media (RPMI 1640 medium, GIBCO) and 0.5% Albumax II (Thermo Fischer Scientific) at 5% hematocrit. The IT4var19 and HB3var03 parasite lines were grown in a gas mixture of 5% O_2_, 5% CO_2_, and 90% N_2_ and 3173-S was grown in a gas mixture of 1% O_2_, 5% CO_2_, and 94% N_2_. All parasites were routinely synchronized by treatment with 5% sorbitol and gelatin flotation to ensure that IEs maintained their ‘knob-like’ adhesion complexes.

### Determination of *var* Gene Transcripts

*P. falciparum*-IEs at ring stage were collected in TRIZOL LS (ThermoFisher Scientific) and RNA was isolated by chloroform and purified with RNeasy Micro Kit (Qiagen), following manufacturer’s instructions. Contaminating genomic DNA was eliminated with DNase I treatment. cDNA was synthesized by reverse transcription reaction. For the IT4var19 and HB3var03 parasite lines, *var* transcription was analyzed by real time-PCR with SYBR green (Power SYBR Green PCR Master Mix, Thermo Fisher Scientific) using a set of IT4var primers (Janes et al., 2011) or HB3var primers (Soerli et al., 2009). Transcription unit levels were normalized to the housekeeping control gene coding for STS (*seryl-tRNA synthetase*). To determine the proportion of 3173-S *var1* transcripts expressed by the 3173-S parasite line, DBLα tags were PCR-amplified using the varF_dg2 and brlong2 primers (Lavstsen et al., 2012), cloned into a Zero Blunt TOPO vector, sequenced, and compared to a previous report (Bernabeu et al., 2019).

### Binding Assay

For IE binding assays, HBMEC, HIMEC and HKMEC were seeded on collagen coated 8 well slides (BD Biocoat) 3–4 days before the assays and allowed to grow to confluency. Mature forms of *P. falciparum* were purified by magnetic purification with LD columns (Miltenyi Biotec) and checked for purity by Giemsa staining. Binding assays and washes were performed with pre-warmed binding medium (RPMI-1640 medium containing 0.5% BSA, pH 7.2). For binding assays, 5x10^6^ IE/ml were resuspended in 200 µl and added to the wells with endothelial cells. After 1 hour incubation at 37°C, non-bound IE were washed by flipping the slides in a gravity wash for 10 min. For binding quantification, slides were fixed in 1% glutaraldehyde for 30 min at room temperature, then stained with 1× Giemsa for 5 min. Binding was quantified in a blinded fashion, by determining the number of IEs adhering per mm^2^ of endothelial cells in six random fields with images taken under 400× magnification (Keyence BZ-X series microscope). For assays with proinflammatory pre-stimulation, confluent cell monolayers were stimulated with 10 ng/ml TNF-α (Sigma, T0157) for 20-24 hours at 37°C prior to the binding assay. For binding inhibition assays, rat anti-human EPCR monoclonal antibody (20 µg/ml, clone RCR-252; Sigma E6280) or rat control IgG (eBiosciences, 20 µg/ml) was added to cells for 30 min of incubation at 37°C prior to the addition of IEs.

### Statistical Analysis

Statistical analysis was performed using Prism (version 8, GraphPad Software Inc.). Flow cytometry data were compared using Two-way ANOVA, Sidak’s multiple comparison test. Binding assays were compared using Two-tailed Unpaired T test. Flow cytometry assays shows the mean of two technical replicates from three or four different experiments. Binding assays with *P. falciparum*-IEs were performed with two technical replicates and conducted in three to six independent experiments.

## Results

### Expression of CD36, ICAM-1, and EPCR on Brain, Intestinal, and Kidney Endothelial Cells

To investigate parasite tropism for brain, gut, and kidney microvascular sites, we first characterized the expression of CD36, ICAM-1 and EPCR on CD31^+^ primary human microvascular endothelial cells. Malaria autopsy studies have indicated that parasite sequestration is higher in the peritubular than the glomerular capillaries (Nguansangiam et al., 2007). Therefore, we compared endothelial cells from brain (HBMEC), intestine (HIMEC) and kidney peritubular (HKMEC) sites by flow cytometry. Cells were compared under resting conditions or following overnight stimulation with TNF-α (see gating strategies in **Supplementary Figure 1**).

**Figure 1:**
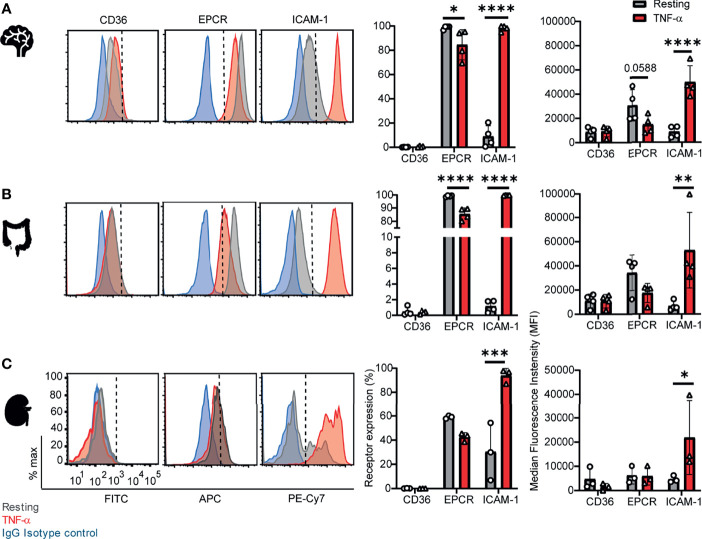
Surface expression of CD36, EPCR, and ICAM-1 on primary human brain, intestinal and kidney endothelial cells. **(A)** Brain, **(B)** Intestinal, or **(C)** Peritubular kidney microvascular endothelial cells were stained for CD36, EPCR and ICAM-1 expression on resting or TNF-α-stimulated cells (20-24 hours treatment). Histograms show expression levels on live/CD31^+^ cells. The percentage of each receptor expression was determined by subtracting isotype control antibody from the target antibody levels. Positive is defined as being above the vertical dashed line in the histogram. Data in bar graphs are expressed as mean ± SD and were analyzed by 2-way ANOVA, using Sidak’s multiple comparisons test. *p<0.05; **p<0.01; ***p<0.001; ****p<0.0001. Additional data is shown in **Supplementary Figures 1, 2**.

Of the three receptors, CD36 had very low expression levels on HBMEC and HIMEC and negligible or absent expression on HKMEC under both resting and TNF-α treatment conditions (**Figure 1**). EPCR had the broadest constitutive expression (100% of HBMEC and HIMEC cells and ~60% of HKMEC cells) and was slightly downregulated on HBMEC and HIMEC following TNF-α treatment (**Figure 1**). By comparison, ICAM-1 had very low constitutive expression levels on a minority of cells (HBMEC, 10%; HIMEC, 1-2%; HKMEC, 10-57%), but its expression was substantially increased by TNF-α treatment reaching nearly 100% surface positive for all three endothelial cell types (**Figure 1** and **Supplementary Figure 2**). Whereas resting EPCR surface levels were higher in HBMEC and HIMEC than HKMEC, constitutive ICAM-1 expression was highest in HKMEC (**Figures 1A, B**). The distinctive expression of the two receptors in HKMEC may be because these cells were cultured in medium supplemented with high concentrations of VEGF to enhance their growth (Ligresti et al., 2016) and VEGF induces ICAM-1 expression on endothelial cells (Kim et al., 2001). Overall, there were similarities in receptor profiles between the three endothelial cell types, especially between the intestinal and brain endothelial cells.

### A Parasite Line Expressing a DC8-PfEMP1 Binds to HIMEC and HKMEC and Is Partially Dependent on EPCR

**Figure 2:**
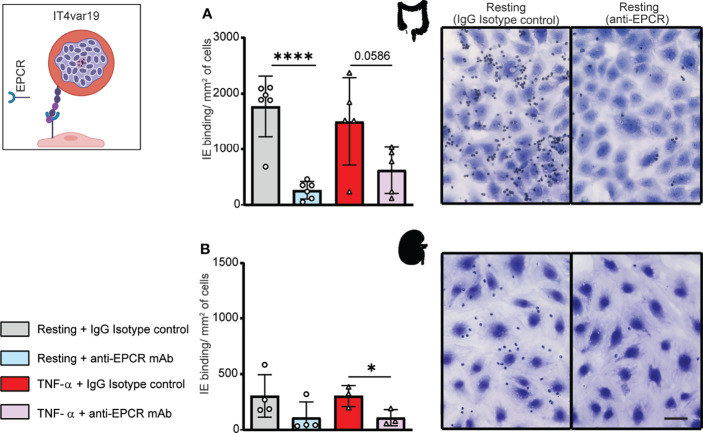
Binding of the IT4var19 clonal parasite line to HIMEC and HKMEC. The IT4var19 parasite was previously selected on brain endothelial cells *in vitro* and expresses a DC8-PfEMP1 protein that interacts with EPCR on endothelial cells (cartoon). **(A)** Binding of IT4var19-IEs to resting and TNF-α-stimulated HIMEC cells in the presence or absence of anti-EPCR antibody. **(B)** Binding of IT4var19-IEs to resting and TNF-α-stimulated HKMEC cells in the presence or absence of anti-EPCR antibody. Data are expressed as mean ± SD and were analyzed by unpaired t test. *p<0.05; ****p<0.0001. EPCR: endothelial protein C receptor. Scale bar: 50 µm. Additional data is shown in **Supplementary Figure 3**.

We next evaluated binding of the IT4var19 clonal line to HIMEC and HKMEC cells. The IT4var19 parasite line expresses a DC8-PfEMP1 variant and was originally selected by repeated panning on an immortalized human brain endothelial cell line followed by limited dilution cloning (Avril et al., 2012). From previous work, IT4var19 is partially dependent on EPCR for binding to brain endothelial cells (Turner et al., 2013; Sampath et al., 2015; Azasi et al., 2018; Bernabeu et al., 2019). The predominant expression of the *var*/PfEMP1 of interest was confirmed by RT-PCR using *var* strain-specific primers for the IT4 parasite genotype (**Supplementary Figure 3**). IT4var19-IEs bound at a slightly higher level to resting than TNF-α-activated HIMEC, albeit it did not reach statistical significance (**Figure 2A**). Binding to both resting and TNF-α-activated cells was substantially inhibited by anti-EPCR monoclonal antibody (median inhibition 80% on resting HIMEC, p<0.0001; median inhibition 60% on TNF-α-activated HIMEC, p = 0.06) (**Figure 2A**). The lower inhibition of anti-EPCR antibodies on activated cells may be due to the reduced EPCR surface expression levels following TNF-α treatment (**Figure 1**).

Likewise, IT4var19 parasites bound to both resting and TNF-α-stimulated HKMEC cells (**Figure 2B**), but at an approximately 6-fold lower level compared to HIMEC (panels A and B in **Figure 2**). The reduced binding levels to HKMEC may be due in part to their lower EPCR expression levels (**Figure 1**), as the interaction of IT4var19 parasites and HKMEC was substantially inhibited by anti-EPCR monoclonal antibody on both resting (median inhibition 70%) and TNF-α-stimulated cells (median inhibition 60%, p<0.05) (**Figure 2B**). Thus, the DC8-PfEMP1 expressing IT4var19 parasite line had binding activity for intestinal and kidney peritubular endothelial cells and is partially dependent on EPCR for adhesion to both cell types, similar to previous findings with primary human brain microvascular endothelial cells (Bernabeu et al., 2019).

### A Parasite Line Expressing a Group A-PfEMP1 Binds to HIMEC and HKMEC

We next investigated a parasite line expressing a Group A PfEMP1 variant. The HB3var03 parasite line was originally selected by repeated panning on an immortalized human brain endothelial cell line (Claessens et al., 2012) and encodes dual binding activity for EPCR and ICAM-1 (Avril et al., 2016; Lennartz et al., 2017; Bernabeu et al., 2019). At the time of this study, the HB3var03 parasite line expressed a mixture of four predominant *var* genes, including the *HB3var03* gene of interest (Group A, dual EPCR + ICAM-1 binder), two Group C predicted CD36 binders (*HB3var29* and *HB3var32*) and a chondroitin sulfate A binder (*HB3var2CSA* allele A) (**Supplementary Figure 4**).

**Figure 3:**
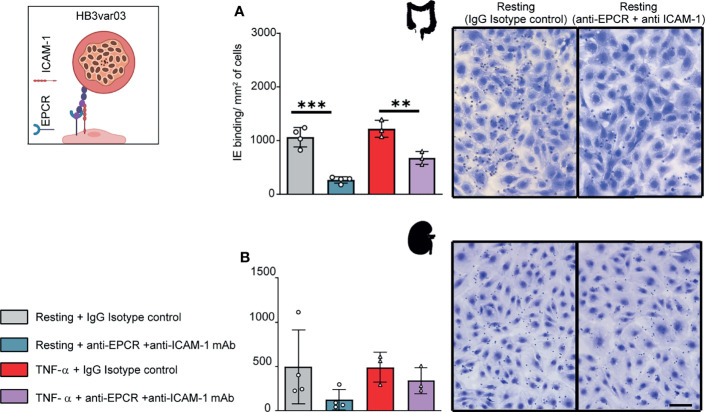
Binding of the HB3var03 parasite line to HIMEC and HKMEC. The HB3var03 parasite was previously selected on brain endothelial cells *in vitro* and expresses a Group A-PfEMP1 protein that interacts with EPCR and ICAM-1 (cartoon). **(A)** Binding of HB3var03-IEs to resting and TNFα-stimulated HIMEC cells in the presence or absence of combined anti-EPCR and anti-ICAM-1 antibodies **(B)** Binding of HB3var03-IEs to resting and TNF-α-stimulated HKMEC cells in the presence or absence of combined anti-EPCR and anti-ICAM-1 antibodies. Data are expressed as mean ± SD and were analyzed by unpaired t test. **p<0.01; ***p<0.001. EPCR: endothelial protein C receptor. Scale bar: 50 µm. Additional data is shown in **Supplementary Figure 4**.

The HB3var03 parasite line bound at a slightly higher level to TNF-α-activated HIMEC than resting cells, albeit it did not reach statistical significance (**Figure 3A**). Because the HB3var03 PfEMP1 encodes dual adhesion properties for EPCR and ICAM-1, the higher binding may be due to the substantial increase in ICAM-1 expression and minor reduction in EPCR surface levels on activated cells (**Figure 1**). Consistent with this interpretation, binding to both resting and TNF-α-activated cells was substantially inhibited by combined anti-EPCR and anti-ICAM-1 monoclonal antibody treatment (median inhibition 75% on resting HIMEC, p<0.001; median inhibition 45% on TNF-α-activated HIMEC, p < 0.01) (**Figure 3A**).

Likewise, HB3var03 parasites bound to both resting and TNF-α-stimulated HKMEC cells (**Figure 3B**). The binding interaction with resting HKMEC was substantially inhibited by combined anti-EPCR and anti-ICAM-1 monoclonal antibody treatment (median inhibition 75%) and less so on TNF-α-stimulated cells (median inhibition 31%) (**Figure 3B**). Like IT4var19, the HB3var03 parasite line had lower binding to HKMEC than HIMEC (panels A and B in **Figure 3**). Taken together, this analysis suggests that Group A PfEMP1 variants with dual EPCR and ICAM-1 binding activity have affinity for intestinal and kidney peritubular endothelial cells.

### A DC8-PfEMP1 Expressing Cerebral Malaria Isolate Binds to Brain, Intestinal and Kidney Endothelial Cells

**Figure 4:**
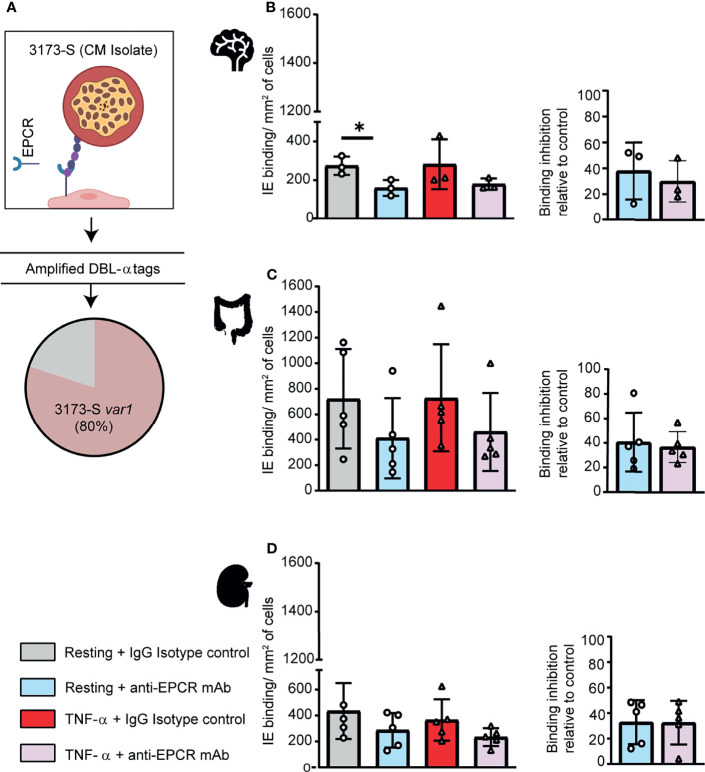
A DC8-PfEMP1 expressing parasite line (3173-S) isolated from a cerebral malaria patient binds to brain, intestinal and kidney endothelial cells**. (A)** The 3173-S parasite line predominantly expresses the *3173-S var1* transcript (DC8-PfEMP1) based on sequencing of DBLα sequence tags. Binding of 3173-S was compared to primary human microvascular endothelial cells from **(B)** brain, **(C)** intestinal, and **(D)** peritubular kidney endothelial cells. Binding levels were compared on resting and TNFα-stimulated cells in the presence and absence of anti-EPCR antibody. Data are expressed as mean ± SD and were analyzed by unpaired t test. *p<0.05. EPCR: endothelial protein C receptor.

To further investigate parasite-endothelial tropism, we evaluated the 3173-S parasite line that was isolated and cloned by limiting dilution from a retinopathy-positive, pediatric CM patient. 3173-S predominantly expresses a DC8-PfEMP1 variant and was previously characterized for binding to 3D human brain microvessels (Bernabeu et al., 2019). The predominant expression of 3173-S *var1* was confirmed by amplification and sequencing of DBLα tags (**Figure 4A**). The 3173-S parasite line bound to all three endothelial cell types with highest binding levels to HIMEC, followed by HKMEC and HBMEC (**Figures 4B–D**). Binding levels of the 3173-S parasite line were similar on resting and activated cells for all three endothelial cell types. 3173-S binding was partially inhibited by anti-EPCR antibody, not exceeding 40% inhibition in any endothelial cell type (**Figure 4**, p<0.05 on resting brain endothelial cells). Taken together, these data suggest that EPCR partially contributes to 3173-S binding to HBMEC, and possibly to HIMEC and HKMEC cells.

## Discussion

Cytoadhesion of *Plasmodium falciparum*-infected erythrocytes (IEs) to the endothelial lining of blood vessels protects parasites from splenic destruction, but also leads to inflammation, vessel occlusion, and organ damage (Miller et al., 2002). Whereas previous work has established that EPCR and ICAM-1 are receptors on brain endothelial cells (Turner et al., 2013; Avril et al., 2016; Lennartz et al., 2017; Bernabeu et al., 2019; Storm et al., 2019), little is known about parasite tropism for gut and kidney, even though multiorgan complications affecting the brain and kidney are common in severe malaria (Dondorp et al., 2008b; Batte et al., 2021) and the gastrointestinal tract is a major site of sequestration in high burden parasite infections (Dondorp et al., 2008a; Milner et al., 2014). To address this knowledge gap, we studied whether parasite lines with affinity for primary human brain endothelial cells would bind to primary microvascular endothelial cells from kidney and gut.

Although CD36 binding is the most common of the PfEMP1 adhesion traits and is encoded by up to 80-85% of proteins in each parasite’s *var* gene repertoire (Robinson et al., 2003; Rask et al., 2010; Smith et al., 2013), this receptor was expressed at very low or negligible levels on brain, intestinal, and peritubular kidney endothelial cells. From histology, CD36 is strongly expressed on liver, spleen, lung, and muscle blood vessels, but is low or absent on brain and kidney endothelial cells (Turner et al., 1994). Together, these findings suggest that CD36 is not widely distributed in the microvascular beds of vital organs, such as brain and kidney, impacted by *P. falciparum* sequestration. The pulmonary vasculature is a site of sequestration (Spitz, 1946; Milner et al., 2015), so a role of CD36 in parasite-lung tropism cannot be excluded.

By comparison, EPCR and ICAM-1 binding domains are encoded by a smaller proportion of PfEMP1 variants, but EPCR had the broadest constitutive expression of the three receptors and ICAM-1 had very low basal expression and was essentially expressed by all brain, gut, and peritubular kidney endothelial cells after TNF-α stimulation, leading to striking differences in receptor profiles between resting (CD36^-^, EPCR^+^, ICAM-1^-/very low^) and TNF-α stimulated endothelial cells (CD36^-^, EPCR^+^, ICAM-1^+^). In line with these *in vitro* findings, ICAM-1 is widely upregulated on microvascular endothelial cells during symptomatic malaria infections (Turner et al., 1994). This study is based on primary endothelial cells. Although nothing is known about receptor expression profiles in the intestine, there is good agreement in CD36 and ICAM-1 expression observed here in primary brain and peritubular kidney endothelial cells and *in vivo* findings (Turner et al., 1994). Overall, our analysis suggests very similar receptor profiles for these three major parasite adhesion receptors on primary human brain, intestinal, and peritubular kidney microvascular endothelial cells.

PfEMP1 proteins have diverged in binding activity for CD36 and EPCR (Smith et al., 2013). Whereas previous work has linked EPCR or dual EPCR and ICAM-1 PfEMP1 variants to severe malaria (Lavstsen et al., 2012; Turner et al., 2013; Bernabeu et al., 2016; Kessler et al., 2017; Storm et al., 2019; Sahu et al., 2021; Wichers et al., 2021), little is known about their endothelial binding specificity. Here, we provide evidence that the DC8 and Group A EPCR-binding PfEMP1 subsets have binding activity for brain, gut, and kidney endothelial cells. Our findings do not preclude that other PfEMP1 variants can sequester in these organs, but they raise the possibility of brain-gut-kidney binding axis contributing to multi-organ complications in severe malaria. A limitation of this study is that binding was studied using cell monolayers and static binding assays. Further work is needed to model parasite binding in organ-specific microvasculature models that better mimic the distinct 3D architecture and microfluidic dynamics of different organs where *P. falciparum*-IEs sequester. Nevertheless, as EPCR-binding variants can inhibit the pro-homeostatic and pro-barrier functions of EPCR by blocking its interaction with the native ligand (Turner et al., 2013; Gillrie et al., 2015; Lau et al., 2015; Petersen et al., 2015; Sampath et al., 2015), multiple organs may be impacted at high parasite burdens.

## Conclusion

This study provides evidence that DC8 and Group A EPCR-binding PfEMP1 variants have broad affinity for brain, intestinal, and kidney microvascular endothelial cells and raise the possibility that a parasite brain-gut-kidney binding axis may contribute to multi-organ dysfunction in severe malaria.

## Data Availability Statement

The original contributions presented in the study are included in the article/**Supplementary Material**. Further inquiries can be directed to the corresponding author.

## Ethics Statement

HKMEC were obtained after voluntary pregnancy interruptions performed at the University of Washington Medical Center in compliance with Institutional Review Board protocol (IRB447773EA).

## Author Contributions

LO, MA, and JS conceived and designed the study. KS provided the parasite line recovered from a pediatric cerebral malaria patient. JX and YZ provided HKMEC cells and culture conditions. LO and MA performed experiments and data analysis. LO and JS wrote the first draft. All authors contributed to the article and approved the submitted version.

## Funding

This work was supported by RO1 AI141602 (JS and KS), U19AI089688 (JS), and UG3/UH3 TR002158 (YZ). The content is solely the responsibility of the authors and does not necessarily represent the official views of the National Institutes of Health.

## Conflict of Interest

The authors declare that the research was conducted in the absence of any commercial or financial relationships that could be construed as a potential conflict of interest.

## Publisher’s Note

All claims expressed in this article are solely those of the authors and do not necessarily represent those of their affiliated organizations, or those of the publisher, the editors and the reviewers. Any product that may be evaluated in this article, or claim that may be made by its manufacturer, is not guaranteed or endorsed by the publisher.
