# Supplementary material for: Plasmodium falciparum Parasite Lines Expressing DC8 and Group A PfEMP1 Bind to Brain, Intestinal, and Kidney Endothelial Cells
Source: Front Cell Infect Microbiol. 2022 Jan 28;12:813011. doi: 10.3389/fcimb.2022.813011 (PMC8831842; doi:10.3389/fcimb.2022.813011)
Supplement: Supplementary file 1 [file Image_1.pdf]

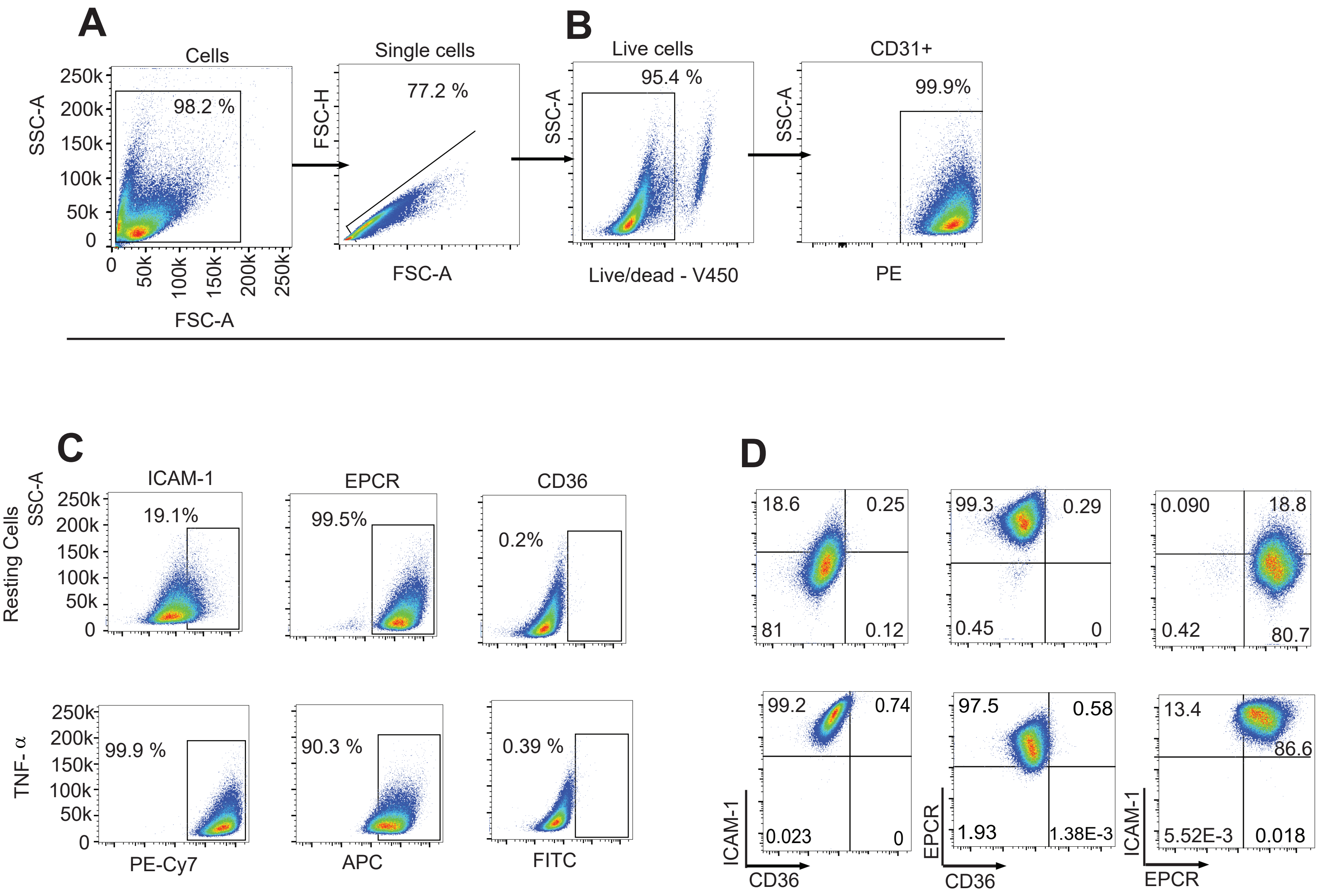

Supplementary Figure 1. Representative flow cytometry gate strategies used for analyzing primary endothelial cell populations. (A) Representative dot plot showing the gate used to identify the single cell population based on foward scatter (FSC) vs. side scatter (SSC) optical detectors. (B) Live cells were gated based on Live/Dead staining (Live/Dead-) and subsequently gated for CD31+ cells (PE). (C) The CD31+ cell population were used to analyze the surface receptors: ICAM-1, EPCR and CD36 based on SSC and the respective secondary antibodies (PE-Cy7, APC and FITC-labeled). (D) Representative dot plot showing cells double-stained for ICAM-1, CD36 and EPCR. EPCR: endothelial protein C Receptor. ICAM-1: intercellular adhesion molecule 1.
