# Supplementary material for: Plasmodium falciparum Parasite Lines Expressing DC8 and Group A PfEMP1 Bind to Brain, Intestinal, and Kidney Endothelial Cells
Source: Front Cell Infect Microbiol. 2022 Jan 28;12:813011. doi: 10.3389/fcimb.2022.813011 (PMC8831842; doi:10.3389/fcimb.2022.813011)
Supplement: Supplementary file 2 [file Image_2.pdf]

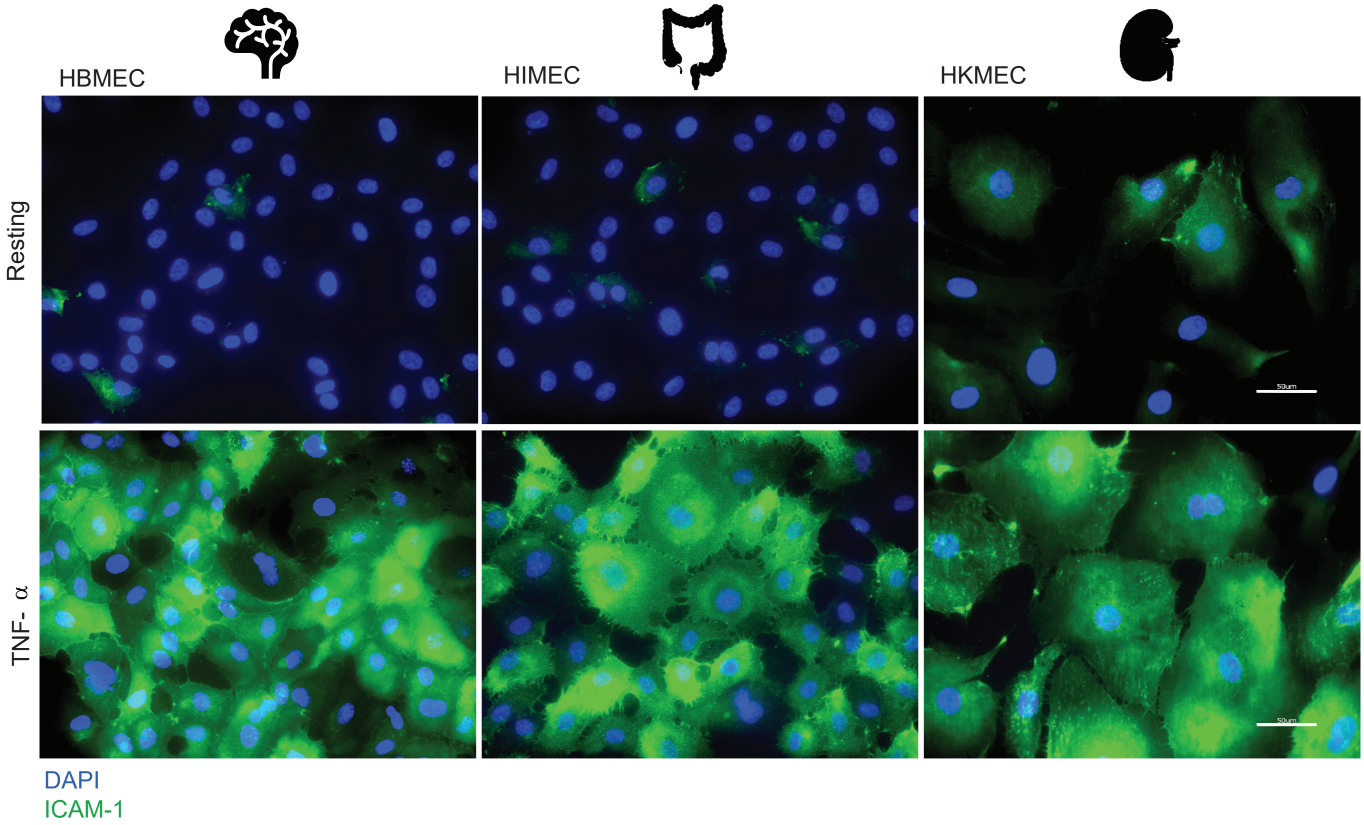


**Supplementary figure 2.** Representative immunofluorescence staining for ICAM-1 (green) merged with DAPI (blue) in brain (HBMEC), intestinal (HIMEC) and kidney (HKMEC) primary endothelial cells under resting or activated conditions (20-24 hours of TNF-α stimulation). Scale bar: 50 μm.
