# Supplementary material for: Plasmodium falciparum Parasite Lines Expressing DC8 and Group A PfEMP1 Bind to Brain, Intestinal, and Kidney Endothelial Cells
Source: Front Cell Infect Microbiol. 2022 Jan 28;12:813011. doi: 10.3389/fcimb.2022.813011 (PMC8831842; doi:10.3389/fcimb.2022.813011)
Supplement: Supplementary file 3 [file Image_3.pdf]

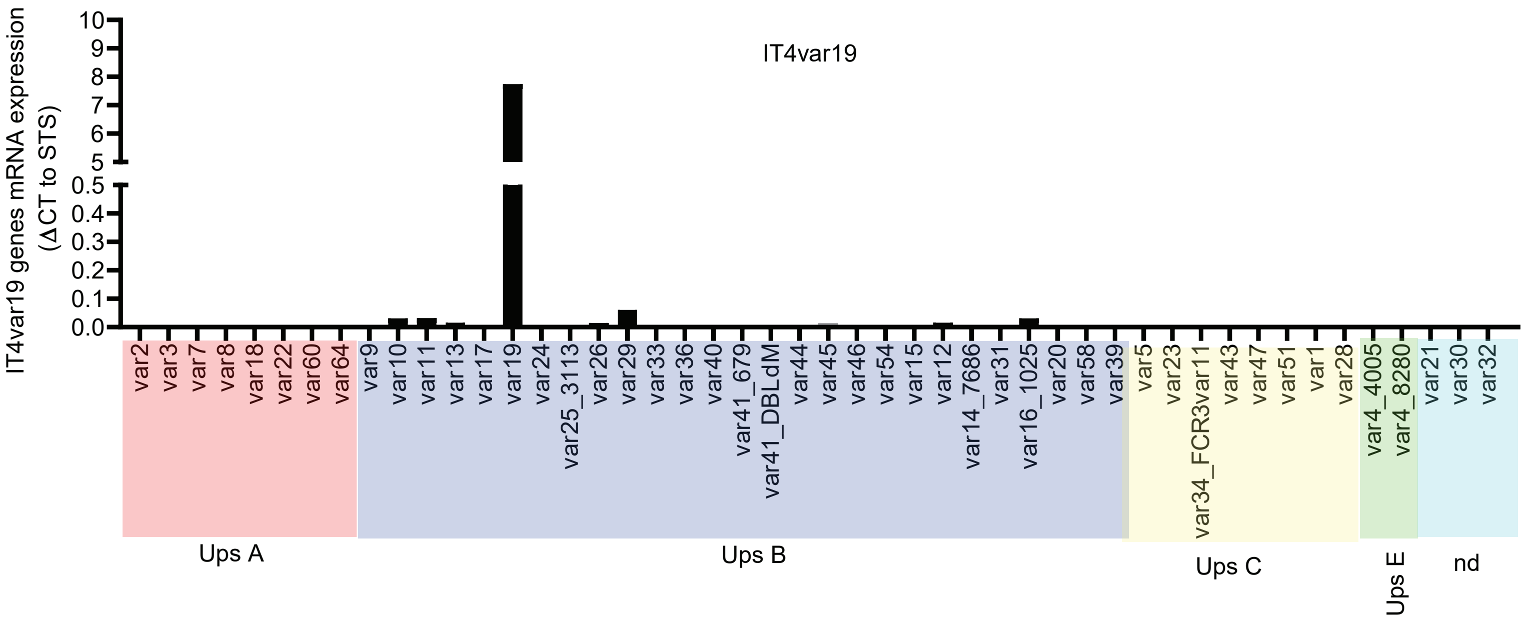

Supplementary figure 3. *var* transcription profile of *P. falciparum* line IT4var19. The *var* gene transcription profile of ring-stage IEs was analyzed by qRT-PCR with IT4 *var* strain-specific primer sets. Transcription unit levels are normalized to the housekeeping control gene coding for STS (seryl-tRNA synthetase). Ups: upstream sequence; nd: not determinated.
